# Intact Pleura during Left Internal Mammary Artery Harvesting in a Patient with kyphoscoliosis and Chronic Obstructive Pulmonary Disease

**Published:** 2014-01-12

**Authors:** Mahmood Hosseinzadeh Maleki, Toba Kazemi, Hamid Reza Mashraghi Moghaddam

**Affiliations:** 1 Mahmood Hosseinzadeh Maleki, Assistant Professor of Cardiac Surgery, Atherosclerosis and Coronary Artery Research Center, Birjand University of Medical Sciences, Valiassr Hospital, Ghaffari Street, Birjand, Iran. 9717853577. Tel: +98 56 1443001-9. Fax: +98 56 14433004. E-mail: mahmoodhosseinzadeh@yahoo.com; 2 Toba Kazemi, Professor of Cardiology,Atherosclerosis and Coronary Artery Research Center, Birjand University of Medical Sciences,Valiassr Hospital, Ghaffari Street, Birjand, Iran. 9717853577.Tel: +98 56 1443001-9. Fax: +98 56 14433004. E-mail: drtooba.kazemi@gmail.com.; 3 Hamid Reza Mashraghi Moghaddam,Assistant Professor of Cardiology, Atherosclerosis and Coronary Artery Research Center,Birjand University of Medical Sciences, Valiassr Hospital, Ghaffari Street, Birjand, Iran. 9717853577. Tel: +98 56 1443001-9. Fax: +98 56 14433004. E-mail: hamid.mashreghi@gmail.com

 We were interested in the study published by Dr. Alizadeh Ghavidel et al.,^1^ whom we congratulate on their publication. There are some points, however, which we wish to make regarding the aim and results of their study.

The left internal mammary artery (LIMA) is mostly used as the conduit of choice for myocardial revascularization. The LIMA confers superior graft patency and better long-term survival and causes fewer cardiac events than does the saphenous vein.^1^^-^^2^ Pleurotomy during LIMA harvesting may cause postoperative events. LIMA harvesting with pleurotomy may affect the respiratory function during the postoperative period, so some surgeons tend to proceed with their operations with the intact pleura.^2^^, ^^3^

With respect to our experience, a 70-year-old male patient with kyphoscoliosis and chronic obstructive pulmonary disease (COPD) presented with post-myocardial infarction angina. He underwent emergent coronary artery bypass grafting (CABG), during which the LIMA was harvested without pleurotomy. A pericardial drain was fixed postoperatively for 6 days, after which time it was removed. It is noteworthy, however, that we ourselves tend to remove the pericardial drain after 2 days. Our patient had a good postoperative period and was discharged on the 10^th^ postoperative day without pulmonary complications. Pre-discharge chest X-ray and echocardiography showed no pleural effusion or pericardial effusion, and post-CABG delayed pericardial effusion was ruled one month later via echocardiography.

We believe that LIMA harvesting without pleurotomy during CABG is associated with lower pulmonary complications, especially in patients with pulmonary comorbidities such as COPD and chest wall deformity. Nevertheless, intact pleura may cause more pericardial effusion and tamponade. We would, therefore, recommend that in these patients, pericardial and retrosternal drains be fixed and kept until pericardial space drainage has dropped to lower than 50 cc per day. Nonetheless, when pleurotomy is done, the drain should be fixed and kept until pericardial space drainage has reduced to lower than 150 cc per day.
