# Supplementary material for: Intact Pleura during Left Internal Mammary Artery Harvesting in a Patient with kyphoscoliosis and Chronic Obstructive Pulmonary Disease
Source: J Tehran Heart Cent. 2014 Jan 12;9(1):52–3. (PMC4277793)
Supplement: Related texts

# Response: Intact Pleura during Left Internal Mammary Artery Harvesting in a Patient with kyphoscoliosis and Chronic Obstructive Pulmonary Disease: Reply

**Published:** 2014-01-12

**Authors:** Yalda Mirmesdagh

**Affiliations:** ***Yalda Mirmesdagh, ****Heart Valve Disease Research Center, Rajaie Cardiovascular, Medical and Research Center, Iran University of Medical Sciences,Niayesh Highway, Valiasr Street, Tehran, Iran. 19969111541. Tel: +98 21 23923061. Fax: +98 21 22663209. E-mail: yalda_2m@yahoo.com.*

 we wish to thank you for your meticulous perusal of our article^1^ and draw your attention to the following points.

Most of the patients candidated for coronary artery bypass grafting (CABG) have some degree of underlying disease because a significant number of these patients are current smokers and are elderly or obese. In addition, it is not uncommon for these patients to suffer from chronic obstructive pulmonary disease (COPD). Underlying pulmonary disease, intubation, prolonged and repeated mechanical ventilation, and infectious complications secondary to repeated or prolonged mechanical ventilation, atelectasis, and pleural effusion may increase treatment costs, length of hospitalization, morbidity, and even mortality. Keeping the pleura intact can be one way to help these patients. The main condition for the preservation of pleural integrity during CABG is precise hemostasis because otherwise the risk of pericardial effusion or tamponade can be problematic, although the incidence of this complication was not significant in our study. 

The next point of significance is harvesting the left internal mammary artery (LIMA) as skeletonized technique. Relevant experiences have indicated that this technique is mostly useful for patients with a short sternum and requires distal coronary artery anastomosis.^2^^-^^4^ Sometimes, small invisible pleural perforations occur during LIMA anastomosis, which may lead to left pneumothorax. In this condition, we recommend that pleural perforation be made (approximately 2 cm) without pleural drain tube insertion and that the sternum be closed after hyperventilation. In these cases, with the aid of mediastinal drainage with negative pressure, pneumothorax may be resolved sooner. Our second recommendation is that, in group of patients subjected to this type of CABG, an extra drain tube such as a chest tube or Tacuson Pratt should be inserted in order to prevent effusion. Also, in order to prevent injury or pressure on the right side of the heart, we can use diaphragmatic pericardium tunneling. 

Finally, based on the results of our article and our personal experiences, we would advise that the pleura be kept intact during CABG. However, the priority is to have a sufficient length of LIMA anastomosis without tension and with proper curve, even though it leads to pleurotomy.

## References

[1] Alizadeh Ghavidel A, Noorizadeh E, Pouraliakbar H, Mirmesdagh Y, Hosseini S, Asgari B, Gholamour Dehaki M (2013). Impact of Intact Pleura during Left Internal Mammary Artery Harvesting on Clinical Outcome. J Teh Univ Heart Ctr.

[2] Raja SG, Dreyfus GD (2005). Internal thoracic artery: to skeletonize or not to skeletonize?. Ann Thorac Surg.

[3] Athanasiou T, Crossman MC, Asimakopoulos G, Cherian A, Weerasinghe A, Glenville B, Casula R (2004). Should the internal thoracic artery be skeletonized?. Ann Thorac Surg.

[4] Wimmer-Greinecker G, Yosseef-Hakimi M, Rinne T, Buhl R, Matheis G, Martens S, Westphal K, Moritz A (1999). Effect of internal thoracic artery preparation on blood loss, lung function, and pain. Ann Thorac Surg.
